# Comment on “Prevalence of Anxiety and Depression in Patients with Inflammatory Bowel Disease”

**DOI:** 10.1155/2018/6747630

**Published:** 2018-12-16

**Authors:** Wenwen Lai, Shaohang Cai

**Affiliations:** ^1^Department of Cardiology, The Second Affiliated Hospital of Fujian Medical University, Quanzhou 362000, China; ^2^Intensive Care Unit, Sun Yat-sen University Cancer Center, Guangzhou 510515, China

We have read with great interest the article presented by Glynis Byrne et al. titled “Prevalence of Anxiety and Depression in Patients with Inflammatory Bowel Disease” published in Canadian Journal of Gastroenterology and Hepatology [1]. The study explored an important but often overlooked area: the mental health status of patients with inflammatory bowel disease (IBD). In the study, the authors found that the rates of depression and anxiety were 25.8% and 21.2% for patients with IBD. Moreover, the results indicated that disease activity was significantly associated with depression and/or anxiety and female patients were more likely to have anxiety. Although the results of the study are interesting and the conclusions are inspiring, the study did not answer some questions and left some blank space in the field. We believe that answering these questions may be more helpful in real clinical practice.

Question one: What causes depression and anxiety in IBD, and what is the change in depression and anxiety after treatment? The relationship between depression and chronic disease is very complicated [2, 3], although research indicates that there is a high morbidity of depression in patients [4]. IBD is an inflammatory disease. Interestingly, it is well established that depression is associated with a chronic, low-grade inflammatory response [5, 6]. For IBD patients, whether depression and anxiety are due to the patient's own self-awareness, or drug-induced, or caused by the disease itself is an unanswered question. Especially for inflammatory disease, the inflammatory response can activate cell-mediated immunity, as well as activation of the compensatory anti-inflammatory reflex system [6]. IBD is often accompanied by an increased serum level of inflammatory factors and superoxide, which contribute to neuropsychiatric disorders [7–9]. The obvious question here is, 'what is the cause of depression and anxiety in IBD?' Furthermore, in this study, the authors found that a significant proportion of patients with IBD have depression and anxiety. What is the change in depression and anxiety after IBD treatment, especially after treatment with nonsteroidal anti-inflammatory drugs? A study previously reported that the state of depression and anxiety can variously be relieved, persist, or be aggravated after treatment for chronic disease [10]. It is currently unknown in IBD research. Comparing changes in anxiety and depression in patients with IBD before and after treatments can evaluate the effectiveness.

Question two: For patients with IBD, do depression and anxiety affect their drug adherence, or their quality of life, or the therapeutic effects? Long-term depression and anxiety can seriously affect a patient's confidence in disease control. Previous researches in chronic diseases suggested that depression and anxiety can reduce patients' drug adherence, resulting in premature drug withdrawals [11, 12]. For IBD patients, regular treatment is very important for controlling disease from progression. Whether depression and anxiety affect drug adherence in patients with IBD has not been reported. In addition, quality of life is one of the important endpoints of IBD treatment. The goal of therapy for IBD is to improve quality of life and survival of the patient by preventing progression of the disease to end-stage disease, colorectal cancer, and death. Whether long-term depression and anxiety affect the quality of life of IBD patients is an unanswered question [12, 13]. Quality of life, as one of the evaluation standards for the effectiveness of treatment, should not be ignored. Finally, whether long-term anxiety and depression affect IBD patients' prognosis is an important but overlooked question. It requires a prospective multicenter randomized controlled trial to confirm.

Question three: Can depression and anxiety be alleviated by psychological intervention in IBD patients? This is a critical issue. It is still not known whether psychological intervention can alleviate anxiety and depression in IBD patients. Previous research suggests that psychological intervention can help relieve depression and anxiety [10]. However, for IBD disease, the causes of anxiety and depression are complicated. Current psychological interventions include health promotion, preventive intervention, psychological counseling, and psychotherapy, while social support includes actively helping patient rebuild social roles, including participation in social organizations (such as sports clubs or entertainment), companionship with friends and family, and help from nongovernmental organizations [14, 15]. What kind of intervention is more effective in relieving depression and anxiety in patients with IBD and whether we can use different interventions depending on the patient's personality traits, so as to obtain a more precision treatment effect, are also unknown, yet.

Nevertheless, the study by Glynis Byrne et al. is an elegant study that gives us a lot of inspiration. Data from this study demonstrate that rates of these mental illnesses would justify screening and referral for psychiatric treatment in clinics. For IBD patients with high-risk factors for depression and anxiety, clinicians should not ignore monitoring their mental state to prevent adverse events.
